# Early warning mechanism for college student adaptation: a network analysis and computational intervention simulation study based on mind wandering and problematic short video use

**DOI:** 10.3389/fpsyg.2026.1739818

**Published:** 2026-03-17

**Authors:** Zheng Mao, Yisheng Yang, Yongzhi Jiang

**Affiliations:** 1 School of Psychology, Inner Mongolia Normal University, Hohhot, China; 2 Inner Mongolia Student Bullying Prevention Research Center, Tongliao, China; 3 School of Educational Science, Inner Mongolia Minzu University, Tongliao, China

**Keywords:** college freshman adaptation, mind wandering, problematic short video use, network analysis, computational simulation, cognitive control, NIRA

## Abstract

**Introduction:**

The adaptation challenges of college freshmen represent a core issue in higher education psychology research. With the rapid development of digital media, this study aims to examine the complex interactions between mind wandering and problematic short video use during the critical period of academic adaptation from a cognitive control perspective.

**Methods:**

A total of 1,989 Chinese college freshmen (*M* = 18.3, *SD* = 0.6) participated in this cross-sectional study. An Ising model network was constructed to estimate the conditional dependency relationships between specific symptoms of mind wandering and problematic short video use. Subsequently, the NodeIdentifyR algorithm (NIRA) was applied to conduct computational intervention simulations to identify the most effective intervention targets within the network.

**Results:**

The network analysis identified two distinct but interconnected symptom communities. Mind wandering during lectures (MW5), inability to pay full attention when doing things (MW3), and difficulty maintaining focus on simple or repetitive tasks (MW1) were identified as key bridge symptoms connecting the two sub-networks. Furthermore, computational simulation results indicated that an alleviating intervention targeting MW5 (reducing its activation level) most effectively decreased the overall severity of problematic short video use.

**Discussion:**

These findings reveal early warning signals of academic adaptation difficulties among college freshmen. By highlighting the pivotal role of specific attention deficits-particularly mind wandering during lectures-this study provides new insights and empirical evidence for designing precise, prevention-oriented interventions in college student mental health education and digital health support.

## Introduction

The college period represents a critical transition from adolescence to adulthood, involving not only significant changes in learning environments and lifestyles but also important reorganization of cognitive, emotional, and social functions (Arnett, 2000). For freshmen entering college campuses, successfully adapting to college life becomes a core challenge affecting academic achievement, mental health, and future development (Credé and Niehorster, 2012). In recent years, with the rapid development of digital media technologies, the adaptation process of college freshmen has presented new characteristics and challenges, particularly as the popularization of short video platforms has profoundly influenced adolescents’ cognitive functions and learning behaviors (Mao et al., 2025a).

### Cognitive perspective on academic adaptation of college freshmen

Academic adaptation is a core dimension of college freshman adjustment, directly related to students’ learning engagement, academic achievement, and mental health (Van Rooij et al., 2018). Compared to high school, college learning requires greater autonomy, involves more complex knowledge structures, and employs more flexible learning methods, which pose unprecedented challenges to students’ cognitive control abilities (Richardson et al., 2012). Research indicates that college freshmen experience varying degrees of academic adaptation difficulties during their first year, manifested as problems with concentration, decreased learning efficiency, and increased procrastination (Tinto, 2012).

From a cognitive neuroscience perspective, successful academic adaptation depends on the effective functioning of the executive function system, particularly core components such as sustained attention, cognitive flexibility, and inhibitory control (Diamond, 2013). However, the prefrontal cortex of adolescents is not yet fully mature, and the executive function system is still developing, making college freshmen more susceptible to cognitive control failures when facing complex learning tasks (Casey et al., 2008). This developmental cognitive vulnerability provides an important neurobiological basis for understanding the academic adaptation difficulties of college freshmen.

### Mind wandering: internal cognitive barrier to academic adaptation

Mind Wandering (MW) refers to the phenomenon where an individual’s attention shifts from the current task or external environment to internal thoughts and imagination, which is a fundamental characteristic of human cognitive activity (Smallwood and Schooler, 2015). Research shows that people spend nearly half of their waking time in a state of mind wandering (Killingsworth and Gilbert, 2010), with this proportion being even higher among adolescents. Although moderate mind wandering may contribute to creative thinking and future planning, excessive mind wandering seriously affects learning efficiency and academic performance (Mooneyham and Schooler, 2013).

The negative impact of mind wandering is particularly prominent in college learning contexts. First, college courses typically require sustained attentional investment and deep information processing, while mind wandering can lead to missing important information and understanding biases (Szpunar et al., 2013a). Second, mind wandering is closely associated with metacognitive monitoring deficits, making it difficult for students to detect when their attention has deviated and thus unable to adjust learning strategies in a timely manner (Szpunar et al., 2013b). Finally, mind wandering may trigger negative emotional cycles, increasing the risk of anxiety and depression, further exacerbating academic adaptation problems (Ottaviani et al., 2013).

### Problematic short video use: new challenges in the digital era

The rise of short video platforms (such as TikTok) has fundamentally changed adolescents’ media consumption patterns and information acquisition methods. These platforms provide highly personalized and attractive content experiences through algorithmic recommendations, immediate feedback, and social interactions (Mao and Jiang, 2023). However, short videos have negative effects on adolescents’ cognitive development and learning behaviors due to their short duration, fast pace, and high stimulation (Mao et al., 2025a).

Problematic Short Video Use (PSVU) refers to excessive dependence and uncontrolled use of short video platforms, characterized by prolonged usage time, difficulty controlling usage impulses, and neglect of other important activities due to usage (Mao et al., 2022). Research shows that at least 11% of adolescents have problems with short video media use (Mao and Jiang, 2022). PSVU not only directly occupies learning time but, more importantly, may damage cognitive functions and learning abilities through multiple mechanisms.

From a cognitive neuroscience perspective, frequent viewing of short videos may lead to fragmentation of attention and decreased cognitive control abilities (Firth et al., 2019). The rapid transitions and high-intensity stimulation of short videos excessively activate the brain’s reward system, reducing interest in traditional offline tasks such as routine learning (Montag et al., 2019). Additionally, adolescents increasingly tend to obtain information through short videos and social media, and this fragmented, passive media usage habit may weaken their deep reading and sustained attention abilities, thus posing challenges to the development of critical thinking and advanced cognitive skills needed in the college stage (Twenge, 2017).

### Potential association between mind wandering and problematic short video use

Although mind wandering and PSVU seem to belong to different categories of psychological phenomena, there may be deep cognitive and neural mechanism connections between them. Both involve failures in attentional regulation and deficits in executive control, with mind wandering reflecting difficulties in endogenous attention control (Huang et al., 2025) and PSVU embodying failures in inhibitory control of external stimuli (Mao et al., 2024). Recent empirical research further shows that mind wandering can positively predict an individual’s PSVU (Huang et al., 2025), providing direct evidence for the above mechanism inference.

From the perspective of network neuroscience, mind wandering and PSVU may share certain abnormal brain network patterns. Research has found that both are associated with functional connectivity abnormalities in the default mode network and salience network (Menon, 2011). This network-level overlap suggests that mind wandering and PSVU may not be independent problems but reflect broader functional disorders of the cognitive control system.

### Intervention strategies for cognitive factors: from traditional approaches to network simulations

Given the central role of cognitive factors in academic adaptation, existing research has explored various intervention strategies targeting attentional control and executive function. Among these, mindfulness meditation is considered one of the most widely studied and effective strategies for mitigating mind wandering. A systematic review by Feruglio et al. (2021) indicated that sustained mindfulness practice significantly reduces the frequency of mind wandering and limits its negative impact on cognitive tasks. Experimental evidence further confirms that mindfulness training not only improves working memory capacity and GRE reading comprehension scores but also enhances task performance directly by reducing distracting thoughts (Mrazek et al., 2013b). Furthermore, cognitive-behavioral interventions targeting attention deficits have shown promise. For instance, the ACCESS program developed by Anastopoulos and King (2015) for college students effectively improved executive function and attention symptoms, while interventions targeting organizational and time management skills have also demonstrated moderate effect sizes in reducing attention symptoms (Hartung et al., 2022).

However, despite the efficacy of these traditional intervention methods, they face several critical limitations that hinder their efficiency in addressing complex adaptation issues. The first is the limitation of “transfer effects.” A meta-analysis by Melby-Lervåg et al. (2016) pointed out that while cognitive training can improve performance on trained tasks (near transfer), evidence for transfer to untrained domains (such as fluid intelligence or broader academic achievement) remains limited, with effect sizes approaching zero under rigorous control conditions. The second limitation involves the ambiguity of “target selection.” Cortese et al. (2015) found that training targeting a single function (e.g., working memory) often fails to improve core symptoms, whereas multi-process training shows better results but makes it difficult to pinpoint which specific component is effective. This “one-size-fits-all” or “trial-and-error” intervention model overlooks the complex interactions between cognitive symptoms, making it difficult to determine which specific symptoms should be prioritized to maximize overall therapeutic outcomes.

To overcome these limitations of traditional approaches, network analysis methods provide a completely new theoretical perspective and analytical framework. Unlike traditional latent variable models, the network perspective conceptualizes psychological phenomena as networks of interacting symptoms (Robinaugh et al., 2020), emphasizing causal relationships and dynamic feedback processes between symptoms (Borsboom, 2017; Borsboom and Cramer, 2013). This perspective not only identifies core symptoms within the network through centrality indices but, more importantly, enables researchers to predict the potential impact of different strategies before implementation when combined with computational simulation methods based on network models, such as the NodeIdentifyR algorithm (Lunansky et al., 2022). By simulating how activating or inhibiting specific symptoms affects the entire network, we can precisely identify key “bridge symptoms” capable of triggering positive cascading effects. This methodological innovation makes it possible to screen for the most cost-effective intervention targets from complex symptom networks, thereby providing precise theoretical guidance for resolving cognitive barriers in college student academic adaptation.

### Research purposes and innovation

Based on the theoretical background above, this study aims to use network analysis and computational intervention simulation methods to deeply explore the network relationship mechanisms between mind wandering and problematic short video use during the academic adaptation process of college freshmen. Specifically, this study attempts to answer the following key questions: (1) How do mind wandering and problematic short video use interact with each other to form what kind of network structure? (2) Which specific mind wandering symptoms play key roles in connecting and maintaining problematic short video use? (3) Through computational simulation, which symptom interventions can most effectively improve problematic short video use?

The innovation of this study is mainly reflected in three aspects: First, innovation in research perspective—for the first time, from the cognitive perspective of academic adaptation, integrating mind wandering and problematic short video use, two seemingly independent but potentially deeply connected phenomena. This integrated perspective helps to more comprehensively understand the adaptation challenges faced by college freshmen. Second, innovation in methodological techniques—adopting network analysis based on the Ising model and computational intervention simulation using the NodeIdentifyR algorithm. This method can capture complex relationships between symptoms and predict intervention effects, going beyond traditional correlation or regression analyses. Third, innovation in practical application—by identifying key intervention targets, providing precise and personalized intervention strategies for college mental health education and academic guidance, which has important practical guiding significance.

## Materials and methods

### Participants and procedures

This study recruited 2,000 Chinese college freshmen through convenience sampling from two comprehensive universities. These students had just completed their high school education and were at a critical transition point from high school to college. The sample represented a diverse range of academic disciplines, covering humanities, social sciences, natural sciences, and engineering. After excluding 11 questionnaires with missing information, a final effective sample of 1,989 was obtained. Participants ranged in age from 17 to 21 years (*M* = 18.3, *SD* = 0.6), with 1,039 males (52.2%) and 950 females (47.8%). All participants were first-time college students who had been enrolled for no more than 3 months.

Regarding the inclusion of students with special needs, this study did not establish disabilities or special educational needs (e.g., Attention Deficit Hyperactivity Disorder, learning disabilities) as specific exclusion criteria; thus, the sample may include students with undiagnosed or undisclosed special needs. However, specific identification data regarding special needs were not collected in the demographic section. This design choice was based on two primary considerations. First, while the study focuses on attentional control mechanisms, the objective was to examine the universal mechanisms linking mind wandering and problematic short video use within the general college population rather than limiting the scope to clinical diagnostic groups. The scales employed in this study measure continuous cognitive and behavioral traits, making them suitable for assessing symptom manifestations across varying degrees of severity. Second, considering the cultural context, research indicates that within Chinese higher education, students are often reluctant to disclose disability status due to concerns regarding the stigma associated with mental health-related disabilities (Kilpatrick et al., 2017; Zhang et al., 2018). Consequently, relying on self-reports might not yield accurate data on special needs and could potentially increase psychological distress for participants.

Data collection followed the ethical guidelines of the Declaration of Helsinki and was approved by the ethics committee of the researchers’ university. Before data collection, trained research assistants explained the research purpose, content, and procedures to the students in detail, emphasizing the voluntary nature of participation and the anonymity of data. All participants signed informed consent forms. Data were collected through an online questionnaire platform.

### Measures

#### Mind wandering scale

We used the Mind Wandering Scale developed by Mrazek et al. (2013a), with the Chinese version revised by Ju et al. (2016). This scale includes five items measuring the frequency and extent to which individuals’ attention deviates from current tasks during daily activities. It uses a 5-point scoring system (1 = almost never, 5 = almost always), with higher scores indicating stronger mind wandering tendencies. The Cronbach’s α coefficient for this scale in the current study was 0.80.

#### Problematic short video use scale

We used the Problematic Short Video Use Scale developed by Mao et al. (2022). This scale is constructed based on the bio-psycho-social model and includes three dimensions: behavioral changes, physiological discomfort, and social adhesiveness, with a total of 13 items. The scale comprehensively assesses the negative impacts of short video use on individual behavioral patterns, physical health, and social functioning. It uses a 5-point scoring system (1 = almost never, 5 = almost always), with higher scores indicating more severe problematic use. Research has shown that this scale has good reliability and validity in both high school and college student populations (Mao et al., 2025b). The Cronbach’s α coefficient for the total scale in this study was 0.87.

### Data analysis

All statistical analyses were conducted using R version 4.5.1 in the RStudio environment. This study employed a two-step analysis strategy: first constructing an Ising model network, then performing computational intervention simulation analysis.

In the first step, we used the Ising model to construct the network structure of mind wandering and problematic short video use (Marsman et al., 2018). The Ising model is a network analysis method suitable for binary data that can estimate conditional dependency relationships between symptoms. Following previous research conventions (Li et al., 2024; Wang et al., 2025), the original scale scores were converted to binary variables: for both the mind wandering scale and problematic short video use scale, scores of 1 were coded as 0 (not present), and scores of 2–5 were coded as 1 (present).

In the network, nodes represent individual symptoms, and edges represent partial correlation coefficients between two symptoms after controlling for all other symptoms (Borsboom et al., 2021). Network parameters were estimated using logistic regression methods, obtained by regressing each variable on all other variables (Van Borkulo et al., 2014). The intercepts (thresholds) of logistic regression indicate the tendency of symptoms to be activated, while coefficients (weights) indicate the strength of associations between symptoms.

To identify key symptoms connecting the mind wandering and problematic short video use sub-networks, we calculated four bridge centrality indices: Bridge Expected Influence, Bridge Strength, Bridge Betweenness, and Bridge Closeness (Jones et al., 2021). The reliability of these indices was evaluated through stability analysis.

In the second step, we used the NodeIdentifyR algorithm (NIRA) to conduct computational intervention simulations on the Ising model network (Lunansky et al., 2022). The Ising model employed in this study originates from statistical physics, initially proposed by Lenz (1920) and further developed by his student Ising (1925) to describe the interaction between magnetic particles (spins) in ferromagnetic materials (Marsman et al., 2018). Recently, this model has been adapted into the field of psychopathological network analysis to capture conditional dependencies between binary variables, such as the presence or absence of symptoms (Marsman et al., 2018; Zhang et al., 2025). In psychological networks, the core mathematical principle of the Ising model can be intuitively understood as follows: each node (symptom) has two possible states—active (1) or inactive (0)—and the edge weights between nodes determine how the activation of one symptom influences the probability of activation in neighboring symptoms. The model characterizes network properties through two key parameters: the *threshold parameter*, representing the independent tendency of each symptom to become active, and the *edge weight parameter*, quantifying the strength of conditional associations between pairs of symptoms (Marsman et al., 2018).

This study utilized the NodeIdentifyR algorithm (NIRA) to conduct simulation-based intervention analysis. NIRA simulates intervention effects by systematically altering the threshold parameters of the Ising network (Zhang et al., 2025). Specifically, two types of interventions were modeled: *alleviating interventions* and *aggravating interventions*. These were implemented by decreasing or increasing the threshold of each symptom by two standard deviations, respectively, representing the amelioration or deterioration of symptoms (Zhang et al., 2025).

The operational mechanism of NIRA is based on simulating the dynamic behavior of the symptom network. When we artificially lower the threshold parameter of a specific symptom, it simulates a clinical intervention scenario where therapeutic measures reduce the probability of that symptom’s occurrence (Lunansky et al., 2022; Zhang et al., 2025). To assess the impact of these perturbations, NIRA calculates a *sum score* reflecting the overall state of the dynamic network; a higher sum score indicates a higher level of psychopathology (Zhang et al., 2025). Specifically, NIRA uses an algorithm to generate a large number of samples (typically 5,000 observations) conditional on the given network parameters to approximate the network’s probability distribution (Lunansky et al., 2022). By comparing the change in the network sum score before and after the intervention, the *projected effect* of each potential intervention target can be quantified. The symptom associated with the largest absolute difference in sum scores pre- and post-intervention is identified by NIRA as the most effective intervention target within the network (Lunansky et al., 2022; Zhang et al., 2025).

It is crucial to clarify that NIRA provides a “computational prediction” based on network structure rather than a direct evaluation of specific intervention techniques (Yu et al., 2025). As emphasized by Yu et al. (2025), when simulation results indicate that a symptom has the maximal effect, this reveals the symptom’s structural importance within the network, rather than directly testing the efficacy of a specific technique such as mindfulness meditation or cognitive-behavioral training.

Consequently, researchers increasingly employ computational simulations, such as those implemented by NIRA, to support more precise clinical recommendations. These simulations virtually amplify or reduce specific symptoms to estimate their impact on the broader network (Yu et al., 2025). Therefore, the intervention strategies proposed in the discussion section (including mindfulness training, metacognitive monitoring, and pedagogical improvements) should be understood as theoretically derived recommendations. These combine the critical targets identified by network analysis with intervention methods proven effective for these specific symptoms in existing empirical literature. The core distinction between this approach and traditional methods lies in the precision of target selection: while traditional methods often employ a “scattershot” strategy, network analysis identifies key symptoms with the greatest “radiating effects,” thereby optimizing the allocation of limited intervention resources (Yu et al., 2025).

Furthermore, alleviating and aggravating interventions hold distinct clinical implications. Alleviating interventions simulate a therapeutic context, answering the question: “Targeting which symptom yields the greatest reduction in overall pathology?” (Yu et al., 2025). Conversely, aggravating interventions simulate risk exposure, revealing which symptoms act as “vulnerable points” that, if exacerbated, would trigger the most significant network-wide deterioration. Research suggests that the same symptom may possess different levels of network influence depending on the direction of the intervention (Yu et al., 2025).

By comparing the changes in total network scores before and after intervention, we identified target symptoms that have the most influence on improving or worsening overall symptoms (Dalege et al., 2017). *T*-tests were used to assess the statistical significance of intervention effects, with FDR method applied for multiple comparison correction.

## Results

### Descriptive statistics

Table 1 presents the descriptive statistics for each symptom of mind wandering and problematic short video use.

**TABLE 1:** Basic information of symptoms and descriptive item statistics.

Symptoms	Items	M	SD	EI
MW1	I have difficulty maintaining focus on simple or repetitive work	2.92	0.75	2.75
MW2	While reading, I find I haven’t been thinking about the text and must therefore read it again	2.74	0.71	1.93
MW3	I do things without paying full attention	2.46	0.71	2.73
MW4	I find myself listening with one ear, thinking about something else at the same time	3.50	0.76	2.29
MW5	I mind-wander during lectures or presentations	2.64	0.78	6.31
PSVU1	Because I use short-form videos too much, I feel indifferent toward the outside world and interact less with others	3.51	0.71	4.40
PSVU2	Once I open a short video app, I find it hard to exit right away and feel compelled to keep watching a bit longer	2.50	0.72	3.09
PSVU3	Short-form videos have trained me to take in a lot of information quickly, and I feel my patience has gotten worse	2.73	0.76	3.43
PSVU4	Frequent or prolonged viewing of short-form videos often leaves me sleep-deprived or with poor sleep quality	3.39	0.70	2.03
PSVU5	Because I often watch short-form videos, I find it hard to get interested in or I resist longer activities	3.41	0.73	1.91
PSVU6	Watching short-form videos makes me sit for long periods and skip exercise, so I often feel tired	3.43	0.76	5.86
PSVU7	Frequent or prolonged viewing of short-form videos often makes my eyes feel dry or my vision feel fatigued	2.81	0.74	2.44
PSVU8	Spending a long time watching short-form videos has reduced the time I spend being physically active	2.52	0.77	11.80
PSVU9	Sitting in the same posture for a long time while watching short-form videos makes my fingers or neck feel sore	3.29	0.68	3.41
PSVU10	I recommend short-form videos I like or find interesting to my friends	2.29	0.71	5.36
PSVU11	I feel that getting information from short form videos is more convenient and efficient	3.21	0.70	4.35
PSVU12	I often receive short-form videos that my friends share with me	3.39	0.72	6.89
PSVU13	Through short-form video platforms, I can promptly get updates about the people I follow	3.25	0.71	5.73

EI, Expected Influence.

### Ising model network structure

**FIGURE 1:**
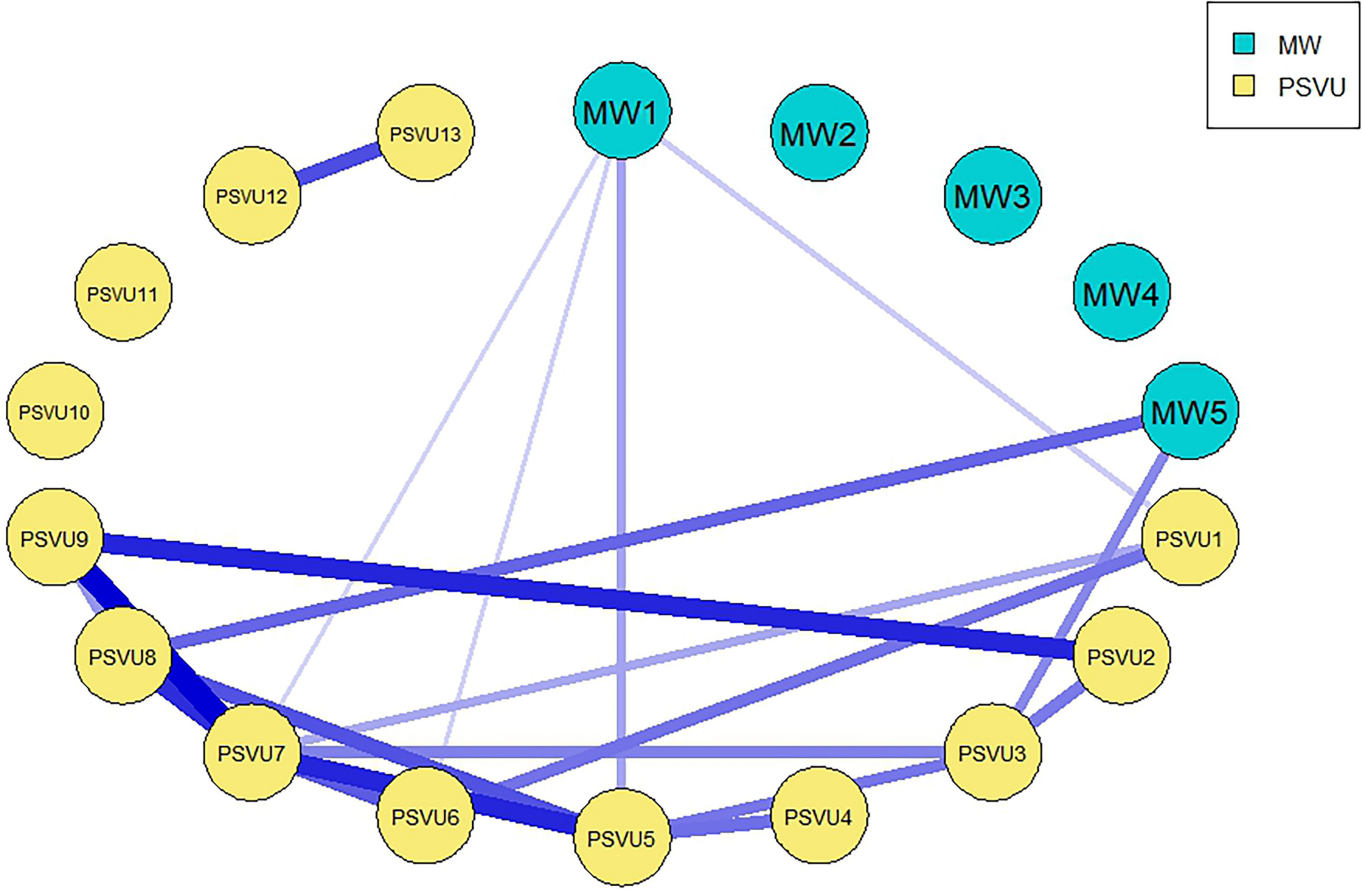
Ising network of MW and PSVU. Edges with weights > 0.1 are displayed in the graph.

Figure 1 shows the network structure of mind wandering and problematic short video use. Network analysis revealed two distinct symptom clusters: the mind wandering sub-network and the problematic short video use sub-network. There are multiple bridge edges connecting the two sub-networks, with the three bridge edges with the largest weights being MW5-PSVU8, MW5-PSVU3, and MW1-PSVU5. Blue edges represent positive correlations, and the thickness of the edges reflects the strength of the association.

**FIGURE 2:**
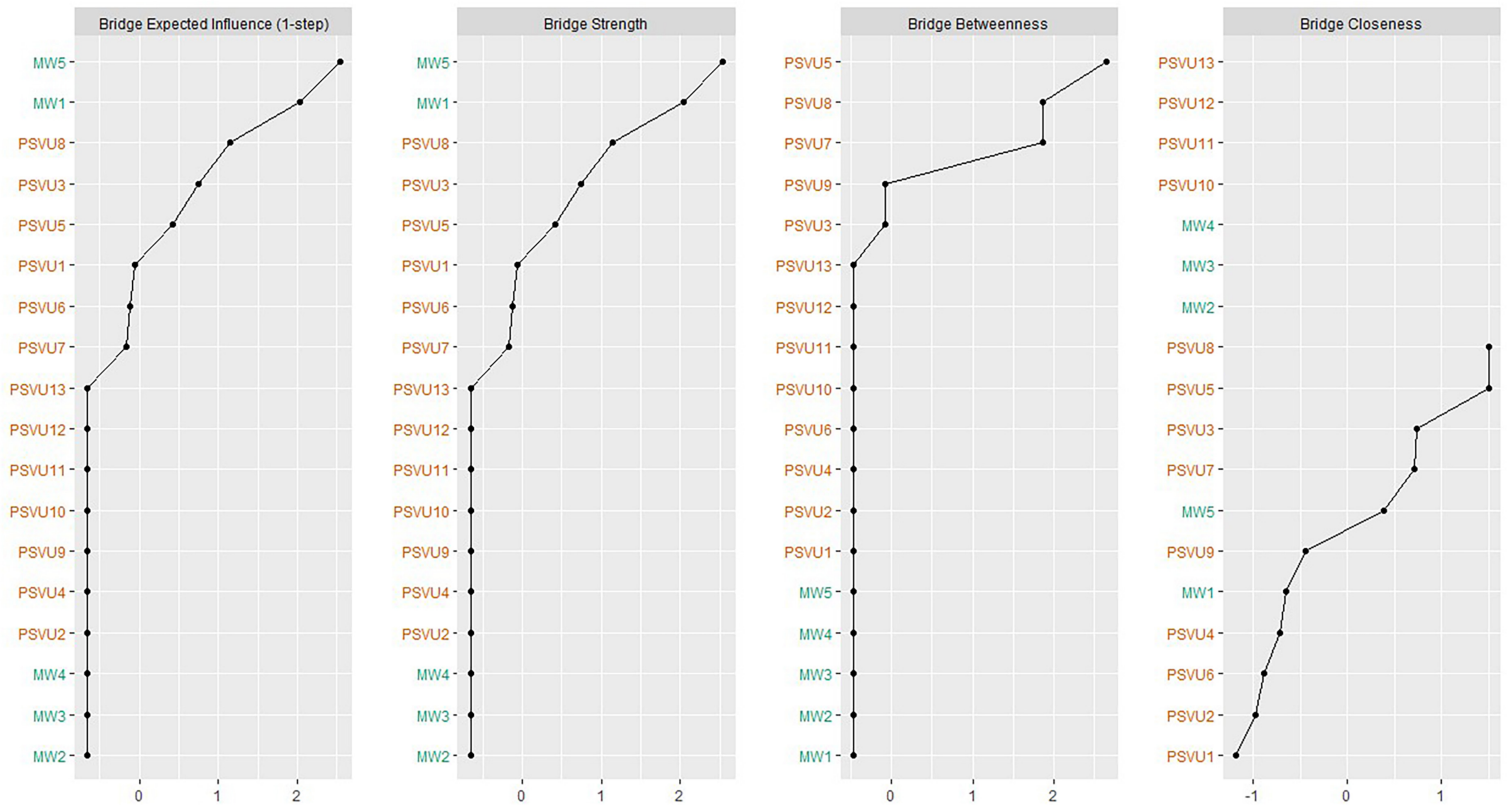
Bridge centrality indices of 18 nodes shown as standardized values z scores.

Bridge centrality analysis (Figure 2) showed that in the mind wandering sub-network, MW1 (difficulty maintaining focus on simple or repetitive tasks) scored highest on the bridge expected influence index, followed closely by MW5 (mind-wandering during lectures) and MW3 (doing things without paying full attention). Stability analysis indicated that bridge expected influence and bridge strength had good stability (CS coefficient > 0.5), while previous research found (Wang et al., 2025) that bridge betweenness and bridge closeness had poorer stability, so the research analysis mainly focused on bridge expected influence and bridge strength indices.

### Computational intervention simulation results

**FIGURE 3:**
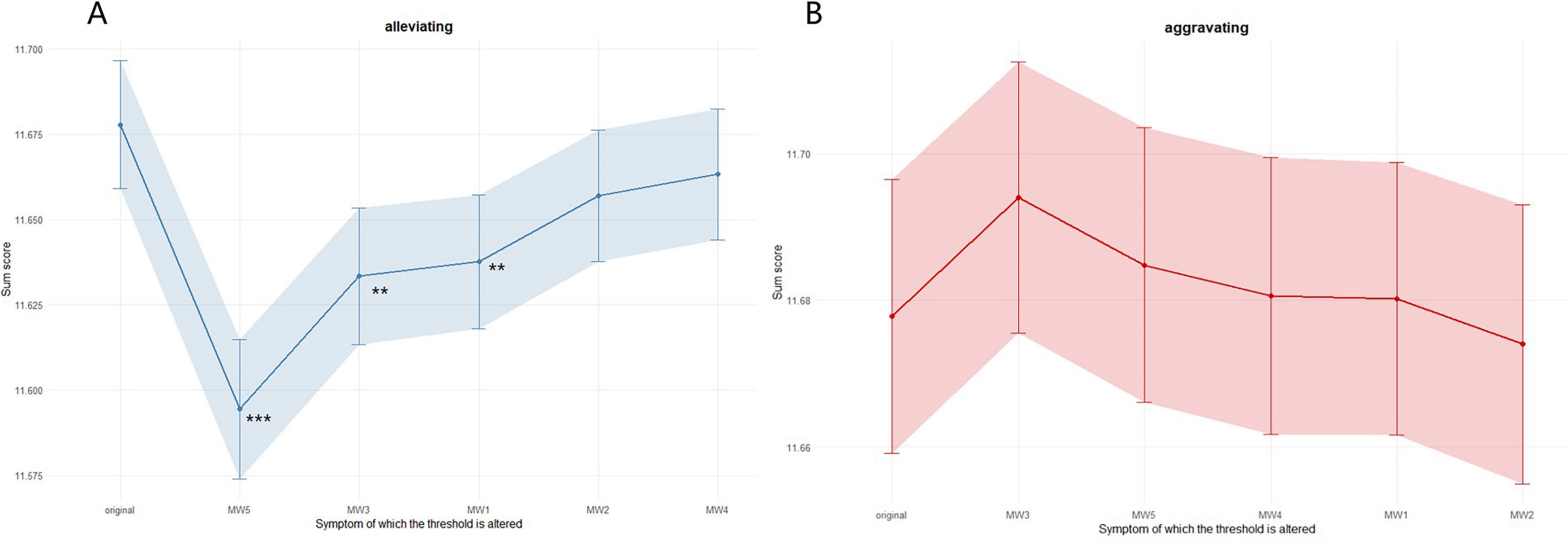
Results of simulating intervention on the Ising model. **(A)** Displays the results of alleviating intervention and **(B)** displays the results of aggravating intervention. Dots represent the network sum scores, while lines represent the 95% confidence interval. The symptoms are listed based on the size of the intervention’s effects, along with the original total score before the intervention. ***p < 0.001, **p < 0.01.

Figure 3 shows the effects of simulated interventions targeting nodes in the mind wandering sub-network. The alleviating intervention results (Figure 3A) showed that reducing the activation level of MW5 (mind-wandering during lectures) had the greatest predictive effect on reducing the total score of problematic short video use. The results indicated that alleviating intervention on MW3 led to a significant decrease in PSVU total score (*t* = 5.91, *p* < 0.001, p.adjust < 0.001). Alleviating interventions on MW3 (doing things without paying full attention) and MW1 (difficulty maintaining focus on simple or repetitive tasks) also showed significant effects (MW3: *t* = 3.18, *p* < 0.01, p.adjust < 0.01; MW1: *t* = 2.91, *p* < 0.01, p.adjust < 0.01).

The aggravating intervention results (Figure 3B) showed that increasing the activation level of mind wandering symptoms had relatively small effects on problematic short video use, with none of the aggravating intervention effects being significant after FDR correction (ps.adjust > 0.05). This asymmetrical result suggests that mind wandering may act more as a susceptibility factor for problematic short video use rather than a direct triggering factor.

## Discussion

Using innovative network analysis and computational intervention simulation methods, this study deeply explored the complex relationship between mind wandering and problematic short video use in the academic adaptation process of college freshmen. The findings not only revealed the internal connection mechanisms between these two types of problem behaviors but, more importantly, identified key targets for precise intervention, providing a new perspective for understanding and improving the adaptation difficulties of college freshmen.

### Methodological note on the terminology of “symptoms”

Before discussing the findings further, it is necessary to clarify the methodological usage of the term “symptoms” in this study. As the reviewers keenly noted, labels such as MW1 are codes for scale items rather than clinical symptom names. This study adopts the term “symptom” following the conventions of network psychopathology. The core tenet of network theory is that symptoms of psychopathology are causally connected through myriad biological, psychological, and societal mechanisms. If these causal relations are sufficiently strong, symptoms can generate a level of feedback sufficient to become self-sustaining. Within the network analysis framework, the term “symptom” carries specific theoretical connotations: it refers not only to symptoms in clinical diagnostic criteria but more broadly to causally active constituent elements within a network (Borsboom, 2017). According to network theory assumptions, symptoms are understood as causally active components of a disorder rather than passive recipients of causal influence (Sobański et al., 2023).

This terminology is grounded in theoretical distinctions. Traditional latent variable models view scale items as passive reflective indicators of a latent construct, assuming that correlations between items stem from a common underlying cause. In contrast, the network perspective reconceptualizes these items as active nodes in a causal network; they do not merely reflect a latent state but, crucially, exert direct mutual influence and dynamic feedback processes upon one another (Borsboom and Cramer, 2013). In the network approach to psychopathology, disorders arise from the causal interplay between symptoms (e.g., worry → insomnia → fatigue), potentially involving feedback loops. For instance, when a student engages in mind wandering in class (MW5), this is not merely a manifestation of a latent trait of “mind wandering tendency,” but an active element in a causal chain that may directly lead to learning difficulties, trigger compensatory short video use, and ultimately exacerbate mind wandering due to cognitive fatigue.

Adopting the term “symptom” facilitates a focus on the core objects of psychopathological research while maintaining consistency with the substantial empirical literature in this field. Network psychopathology approaches are becoming increasingly prevalent in mental health research. This paper illustrates contemporary practices in applying network analysis tools, bridging the gap between network concepts and their empirical application. Since the foundational work of Borsboom and Cramer (2013), using symptoms as network nodes has been widely adopted, as evidenced in numerous studies on depression, anxiety, and PTSD (Contreras et al., 2019). It should be noted that nodes in network analysis can also include traits, affect, behaviors, and cognitions (Briganti et al., 2024); however, this study chooses to focus on the symptom level to engage directly with existing clinical psychopathology literature.

Nonetheless, we acknowledge that this terminology may create ambiguity in non-clinical contexts. Mind wandering and problematic short video use, as continuous cognitive-behavioral features, do differ from symptoms in traditional psychopathology. Therefore, readers should interpret “symptoms” in this study as “operationalized indicators with causal associations within a network,” rather than strictly as clinical symptomatology. This clarification also helps avoid the over-pathologization of the findings; our goal is to understand the cognitive mechanisms underlying common adaptive challenges faced by college freshmen, rather than to label these challenges as psychiatric problems.

### Network characteristics of mind wandering and problematic short video use: dual manifestations of cognitive control dysregulation

The network analysis results clearly showed that mind wandering and problematic short video use form two relatively independent but closely related symptom communities. This network topological structure supports our theoretical hypothesis: although the two belong to different problem categories on the surface, there are substantial connections in deep cognitive mechanisms. This finding is consistent with the dual-system theory of cognitive control (Hofmann et al., 2009), suggesting that the imbalance between the top-down control system (corresponding to mind wandering) and the bottom-up impulse system (corresponding to problematic short video use) may be the core mechanism of academic adaptation difficulties for college freshmen.

Particularly noteworthy is that “mind-wandering during lectures” (MW5) exhibited the most prominent importance in the entire network. This finding has profound theoretical and practical significance. Lectures are the core form of college education, requiring students to maintain sustained attention to complex information over a relatively long period (Lindquist and McLean, 2011). Mind-wandering during lectures not only reflects a failure of advanced cognitive control but may also be a concentrated manifestation of functional disorders of the entire attention system. Unlike simple tasks, lecture comprehension requires simultaneous coordination of multiple cognitive processes: sustained attention, working memory updating, semantic integration, and inhibition of irrelevant information (Smallwood et al., 2007). When this complex cognitive coordination experiences problems, individuals may turn to activities with low cognitive load, such as short videos, for immediate satisfaction.

From a cognitive neuroscience perspective, maintaining attention during lectures involves dynamic balance between the frontoparietal control network and the default mode network (Christoff et al., 2016). Research has shown that the occurrence of mind wandering is closely related to activation of the default mode network (Fox et al., 2015). The cognitive challenges faced by college freshmen may exacerbate this network imbalance, while the algorithmic recommendations and rapid switching characteristics of short videos cause adolescents’ attention to be interrupted, increasing cognitive load and daily stress, thereby forming a state of distracted attention (Kushlev and Dunn, 2015). This immediate but superficial cognitive satisfaction may further weaken students’ ability to maintain deep attention.

### Identification of key intervention targets: from network structure to dynamic intervention

The computational intervention simulation results revealed a key finding: MW5 (mind-wandering during lectures) is the most effective intervention target, with its alleviation producing the greatest network improvement effect. The importance of this result cannot be overlooked. Attention problems during lectures may represent “upstream” damage to the cognitive control system, and its improvement can affect the entire symptom network through cascade effects (Borsboom and Cramer, 2013). Specifically, when students can maintain focus during lectures, they not only directly enhance learning outcomes but may also strengthen academic self-efficacy and reduce avoidant media use behaviors arising from academic difficulties (Zimmerman, 2000).

MW3 (doing things without paying full attention) and MW1 (difficulty maintaining focus on simple or repetitive tasks) also showed significant but decreasing intervention effects. This hierarchical pattern reveals the functional organizational characteristics of the attention system. According to Petersen and Posner’s (2012) attention network theory, MW5 mainly involves advanced functions of the executive attention network, MW3 reflects task-set maintenance capabilities, while MW1 involves more basic alerting maintenance. This gradient of intervention effects from advanced to basic suggests that interventions targeting higher cognitive functions may have more extensive top-down effects, supporting the possibility of “far transfer” effects in cognitive training (Simons et al., 2016).

Particularly noteworthy is that the prominent intervention effect of MW5 may be closely related to the special nature of college learning. Lecture learning is not only the main pathway for knowledge acquisition but also a key scenario for cultivating critical thinking and deep understanding. When students can effectively control mind-wandering during lectures, they may simultaneously improve multiple executive function components such as metacognitive monitoring, cognitive flexibility, and inhibitory control (Szpunar et al., 2013b). This multidimensional cognitive improvement may explain why interventions targeting MW5 can produce such significant network effects.

The general non-significance of aggravating intervention effects reveals the resilient characteristics of symptom networks. Psychological symptom networks may exhibit non-linear responses when facing disturbances (Mao et al., 2025a) and return to their original stable state when not sufficiently destabilized. This system stability can be viewed as a manifestation of a self-stabilizing mechanism, preliminarily explaining why certain interventions fail to induce sustained changes (Hayes et al., 2007). This asymmetry, where alleviating interventions are effective while aggravating interventions are ineffective, emphasizes the critical role of prevention and early intervention, especially during the developmentally sensitive period of college freshmen, where timely attention training may prevent the solidification of problematic behavioral patterns.

### Theoretical contributions: integrating cognitive psychology and digital media research

The theoretical contributions of this study are primarily reflected at three levels. First, the study established theoretical connections between mind wandering and problematic short video use, filling research gaps between cognitive psychology and digital media psychology. This cross-domain integration provides a new framework for understanding adolescent cognitive development in the digital era. Traditionally, mind wandering research has mainly focused on its impact on task performance (Randall et al., 2014), while media overuse research has focused on addiction mechanisms (Griffiths, 2018). This study revealed the common cognitive foundations of both, laying the groundwork for constructing more comprehensive theoretical models.

Second, the study validated the value of network analysis methods in understanding complex psychological phenomena. By conceptualizing symptoms as interactive networks, we can go beyond traditional linear models to capture the emergent properties of psychological problems. This methodological innovation has important implications for understanding psychological phenomena involving multiple interacting factors. The network perspective emphasizes mutual activation and maintenance between symptoms, which is highly consistent with the distributed processing characteristics of cognitive systems (McClelland et al., 2010).

Third, the study provided new evidence for developmental theories of cognitive control. College freshmen are at a critical developmental period for cognitive control systems, and the attention deficit patterns found in this study support the delayed maturation hypothesis of the prefrontal cortex (Gogtay et al., 2004). More importantly, the study revealed how digital media environments interact with developing cognitive systems, which is significant for understanding the cognitive developmental trajectories of contemporary adolescents.

### Practical significance: formulating precise intervention strategies

The research results have direct practical guiding significance for college student mental health education and academic support. First, identifying MW5, MW3, and MW1 as key intervention targets provides clear direction for designing targeted cognitive training programs. Based on these findings, training modules specifically targeting sustained attention, selective attention, and attentional control can be developed.

Specifically, interventions targeting MW5 (mind-wandering during lectures) need to improve students’ metacognitive monitoring abilities. Students can be trained to use “attention check” strategies to regularly self-assess their attentional state and promptly detect and correct mind-wandering (Szpunar et al., 2013b). Meanwhile, improving classroom teaching methods, such as increasing interactive sessions and using concept-checking questions, can help maintain student attentional engagement.

Interventions targeting MW3 (doing things without paying full attention) can adopt cognitive control training. Cognitive training paradigms such as dual n-back tasks and task-switching training have been proven to improve executive functions (Jaeggi et al., 2008). Gamifying these training exercises and integrating them into mobile applications can increase college students’ engagement and training effectiveness.

Interventions targeting MW1 (difficulty focusing on simple repetitive tasks) can adopt mindfulness training and attentional anchoring techniques. Research has shown that mindfulness meditation can enhance sustained attention ability and improve regulation of the default mode network (Lutz et al., 2008). Two weeks of mindfulness training can significantly reduce the frequency of mind wandering and improve task focus (Mrazek et al., 2013b). For college freshmen, simplified versions of mindfulness exercises can be designed, such as the 3-min breathing space practice, which they can quickly apply and benefit from in daily learning.

### Prevention-oriented intervention concept

The finding that aggravating interventions had non-significant effects emphasizes the importance of prevention. Once patterns of problematic short video use are formed, they are difficult to reverse by changing a single cognitive factor. This suggests that colleges should conduct preventive education and screening at the beginning of freshmen enrollment. Early warning systems based on the findings of this study can be developed to identify high-risk students through brief mind wandering assessments and provide timely support.

Prevention strategies should be multi-level. At the individual level, cultivate students’ self-regulation abilities and media literacy; at the environmental level, create learning environments that support focus, such as establishing “phone-free learning zones” and promoting time management techniques like the “Pomodoro Technique”; at the institutional level, incorporate attention training and cognitive skill development into freshman adaptation education courses.

### New ideas for digital health interventions

The research results also provide new ideas for digital health interventions. Since problematic short video use is closely related to attention deficits, “cognitively friendly” short video platform functions can be designed. For example, setting usage time reminders, inserting attention restoration exercises, and providing usage pattern feedback. This “nudge” style of intervention does not require extra effort from users but may produce positive effects (Thaler and Sunstein, 2008).

Furthermore, personalized intervention systems based on artificial intelligence can be developed. By monitoring users’ usage patterns and attentional states, the system can identify risk moments and provide immediate interventions. For example, when detecting that a user has been continuously watching short videos beyond a set time, automatically pushing an attention training mini-game or mindfulness practice audio.

### Limitations and future directions

Despite providing important findings, this study has several limitations that need to be improved in future research. First, the cross-sectional design limits the reliability of causal inferences. Although computational simulations provide indirect evidence of causal relationships, longitudinal research and experimental studies are needed for validation. Future research could conduct multi-time point intensive tracking studies using ecological momentary assessment (EMA) methods to capture the dynamic change processes of mind wandering and short video use.

Second, the sample was limited to Chinese college freshmen, and the universality of the research findings needs further testing. Educational systems, learning requirements, and media usage habits in different cultural backgrounds may affect the relationship patterns between mind wandering and problematic short video use. Cross-cultural comparative research will help identify universal mechanisms and culturally specific factors.

Third, binary coding may lose information about symptom severity. Although the Ising model is suitable for binary data, future research could explore other network models, such as Gaussian graphical models or mixed graphical models, to retain more quantitative information. Meanwhile, different threshold settings could be tried to test the robustness of the results.

Fourth, the study only focused on mind wandering and problematic short video use, but college freshmen adaptation involves broader factors. Future research could incorporate other relevant variables such as academic stress, social anxiety, and sleep quality to construct more comprehensive adaptation difficulty network models. Additionally, how individual difference factors (such as personality traits, cognitive abilities, and family background) moderate network structure and intervention effects is also worth in-depth exploration.

Fifth, this study did not incorporate important contextual variables such as students’ motivation type regarding their chosen major and program-level interest diversity, which constitutes a limitation. Literature indicates that students who choose majors matching their vocational interests achieve better academic results and are more likely to complete higher education on time (Schelfhout et al., 2019; de Vries et al., 2024). According to Holland’s person-environment fit theory, students are more likely to experience higher learning motivation and satisfaction when their vocational interests align with their academic environment. Schelfhout et al. (2019) further found that autonomous motivation is negatively correlated with program interest diversity, whereas controlled motivation is positively correlated with it, suggesting that motivation type may play a crucial role between interest fit and academic outcomes. Based on this theoretical framework, we postulate that this fit may moderate the frequency of mind wandering by influencing students’ motivation types: Students with high autonomous motivation and passion for their major may maintain classroom attention more easily, whereas those driven primarily by external motivation or poor major fit may mind-wander more frequently and turn to short videos as an alternative source of gratification. Future research should consider including variables such as motivation type and program interest diversity to more comprehensively understand the relationship between interest fit and classroom attention.

From the perspective of Self-Determination Theory, academic motivation can be distinguished into autonomous motivation (driven by intrinsic interest and value identification) and controlled motivation (driven by external pressure or rewards) (Deci et al., 2017). Research shows that variance in program interest diversity is associated with autonomous and controlled motivation within student populations (Schelfhout et al., 2019). Students with high autonomous motivation tend to view academic activities as goal-pursuit processes consistent with the self, which is associated with higher levels of academic engagement (Singh et al., 2022). Specifically, autonomous motivation promotes adaptive behaviors in learning (e.g., planning, task management, and persistence) by enhancing students’ identification with learning tasks and mastery orientation (Singh et al., 2022), which may strengthen their ability to resist mind wandering and the temptation of short videos. Academic engagement significantly impacts academic performance and is closely linked to career development (Chen et al., 2023); high engagement levels correlate with better knowledge acquisition, cognitive development, and academic achievement (Singh et al., 2022; Schelfhout et al., 2019). Social support significantly and positively predicts academic engagement, influencing it through the mediating effect of academic motivation (Chen et al., 2023). Conversely, students who choose majors primarily due to parental expectations or employment pressure (i.e., high controlled motivation) may lack the internal resources to cope with academic challenges, indicating low student-major fit (Schelfhout et al., 2019). Such students are more likely to exhibit maladaptive cognitions and behaviors (e.g., anxiety, failure avoidance, and uncertainty), which are significantly negatively correlated with academic engagement (Singh et al., 2022), thereby weakening their ability to cope with academic challenges. The research context involves university students, as there are concerns that motivation may decline as individuals progress through their academic careers (Singh et al., 2022). Future research should consider incorporating major fit and academic motivation types into network models to examine whether they moderate the strength of the association between mind wandering and problematic short video use.

Overall, research on mind wandering in the classroom emphasizes the need for evidence-based studies that consider how classroom instruction is structured and which interventions might effectively maintain student interest and attention. Educational guidelines typically urge teachers to intersperse lectures with tasks that help refocus student attention. Instructional strategies have a direct impact on maintaining classroom attention and reducing mind wandering. Promoting active learning through demonstrations, discussions, or other activities utilizes a range of pedagogical methods to reduce mind wandering; when activity ceases and something new begins, most wandering minds return (Pachai et al., 2016). Szpunar et al. (2013a) provide strong evidence that interpolating quizzes during lectures, as an active learning strategy, can effectively interrupt the chain of mind wandering and improve learning outcomes. Their study indicates that testing sustains attention to lectures by inhibiting task-irrelevant activities (such as mind wandering) and reinforcing task-relevant cognitive engagement (such as note-taking).

This finding has important implications for understanding the results of the present study: the identification of MW5 (mind wandering during class) as the most critical intervention target may reflect the prevalence of passive, unidirectional knowledge transmission in current university teaching. If instructors adopted more active learning methods—including interactive and inquiry-based teaching—the baseline level of classroom mind wandering might decrease, potentially altering the strength of the network relationship between mind wandering and problematic short video use. This effect may stem from the distinction between active and passive states (e.g., active engagement via intermittent testing appears helpful) (Szpunar et al., 2013a), or from the asymmetry in social dynamics between students and teachers. Indeed, recent online learning research suggests that having students teach concepts to virtual students (adopting the teacher’s perspective) improves memory for course content (Szpunar et al., 2013a). Furthermore, career adaptability theory posits that integrating academic content with students’ career goals and curiosity, alongside autonomy-supportive teaching practices, can significantly enhance student academic engagement and satisfaction (Oliveira and Marques, 2024).

Future research should employ multi-level designs to cross-model student-level cognitive characteristics with classroom-level instructional features. For instance, based on social support theory (Chen et al., 2023), researchers could explore how teacher support reduces mind wandering by enhancing students’ life satisfaction and academic motivation, or whether teacher social support strengthens students’ positive emotional experiences, thereby reducing the transition from mind wandering to problematic media use and enabling more effective coping with academic challenges (Chen et al., 2023). Additionally, longitudinal studies could explore whether mind wandering and problematic short video use decrease as students progress deeper into professional training, potentially reflecting enhanced professional identity, increased academic self-efficacy, and deepened interest in professional content. Academic motivation is the internal drive directly propelling student learning, serving initiation, maintenance, and directional functions. The nature and intensity of academic motivation directly influence the direction, process, and outcomes of university students’ learning. Such research would not only reveal dynamic trajectories of academic adaptation but also provide empirical evidence for higher education institutions to optimize major streaming mechanisms and improve teaching quality.

Finally, although NIRA provides valuable information on intervention targets, actual intervention effects still need to be verified through clinical trials. Future research should design intervention programs based on the targets identified in this study and evaluate their effectiveness through randomized controlled trials. At the same time, the optimal intervention dosage, timing, and combination methods need to be explored.

## Conclusion

Through innovative network analysis and computational simulation methods, this study revealed the complex relationship mechanisms between mind wandering and problematic short video use in the academic adaptation process of college freshmen. The research found that these two seemingly independent problems have deep connections at the level of cognitive control, with deficits in basic attentional functions potentially being the key bridge connecting them. By identifying key intervention targets such as “difficulty maintaining focus on simple or repetitive tasks,” the study provides scientific basis for formulating precise early intervention strategies.

These findings not only deepen our understanding of the cognitive developmental challenges faced by adolescents in the digital age but also provide important insights for higher education practice. Facing cognitive challenges brought by rapid technological development, comprehensive intervention strategies that prioritize prevention, focus on cognitive training, and ensure environmental support need to be adopted. Only in this way can we help college freshmen successfully adapt to academic requirements, fully realize their potential, and contribute to personal development and social progress.

Future research should continue to deepen understanding of the relationship between mind wandering and problematic media use, develop and validate intervention programs based on the findings of this study, and explore personalized and intelligent intervention methods. At the same time, it is necessary to examine the impact of digital technology on human cognition and behavior from a broader perspective and provide psychological wisdom for constructing a future society where humans and machines harmoniously coexist.

## Data Availability

The original contributions presented in the study are included in the article/supplementary material, further inquiries can be directed to the corresponding author.
